# p21/WAF1 expression in human colorectal carcinoma: association with p53, transcription factor AP-2 and prognosis

**DOI:** 10.1038/sj.bjc.6690662

**Published:** 1999-09

**Authors:** K M Ropponen, J K Kellokoski, P K Lipponen, T Pietiläinen, M J Eskelinen, E M Alhava, V-M Kosma

**Affiliations:** Department of Pathology and Forensic Medicine, University of Kuopio, PO Box 1627, Kuopio, FIN-70211, Finland; Departments of Surgery, Clinical Pathology Oral and Maxillofacial Unit, Otorhinolaryngology and Oncology and Otorhinolaryngology and Oncology, Kuopio University Hospital, PO Box 1777, Kuopio, FIN-70211, Finland

**Keywords:** colorectal carcinoma, prognosis, p21, p53, AP-2

## Abstract

p21/WAF1 expression was studied in a series of 162 colorectal carcinoma patients and its relation to p53- and activator protein (AP)-2 expressions and to stage as well as survival was assessed. p21 expression was moderate or intense in 33% of the tumours, and 53% of the tumours had moderate or strong p53 staining intensity. Eighty-nine percent of the tumours showed a weak cytoplasmic AP-2 signal. As expected, p21 and p53 stainings were inversely related to each other (*P* < 0.001). There was a significant positive association between p21 and AP-2 expression levels (*P* = 0.01). p21 intensity and percentage were higher in Dukes' A and B stages (*P* < 0.001). The cancer-related survival and recurrence-free survival (RFS) rates were significantly lower among patients with a low signal for p21 (*P* < 0.001) and low p21 percentage in tumour epithelium (*P* < 0.001). High p53 staining intensity in tumour epithelium predicted poor survival (*P* = 0.01) and RFS (*P* = 0.003). In the multivariate analysis, p21 percentage distribution independently predicted cancer-related survival in all cases, and p21 expression intensity in T1–4/N0–3/M0 and T1–3/N0/M0 cases. p21 percentage distribution was an independent predictor of RFS in all and T1–3/ N0/M0 cases. AP-2 staining did not reach any prognostic significance. These results suggest that the immunohistochemical detection of cyclin-dependent kinase inhibitor p21 could be used to predict more precisely the outcome of colorectal cancer patients. © 1999 Cancer Research Campaign


*British Journal of Cancer* (1999) **81**(1), 133–140
© 1999 Cancer Research Campaign
Article no. bjoc.1999.0662

### p21/WAF1 expression in human colorectal carcinoma: association with p53, transcription factor AP-2 and prognosis

**KM Ropponen[1,3], JK Kellokoski[1,4,5], PK Lipponen[1], T Pietiläinen[1,3], MJ Eskelinen[2], EM Alhava[2] and V-M Kosma[1,3]**

[1]Department of Pathology and Forensic Medicine, University of Kuopio, PO Box 1627, FIN-70211 Kuopio, Finland; Departments of [2]Surgery, [3]Clinical Pathology, Oral and Maxillofacial Unit, [4]Otorhinolaryngology and [5]Oncology, Kuopio University Hospital, PO Box 1777, FIN-70211 Kuopio, Finland

**Summary** p21/WAF1 expression was studied in a series of 162 colorectal carcinoma patients and its relation to p53- and activator protein (AP)-2 expressions and to stage as well as survival was assessed. p21 expression was moderate or intense in 33% of the tumours, and 53% of the tumours had moderate or strong p53 staining intensity. Eighty-nine percent of the tumours showed a weak cytoplasmic AP-2 signal. As expected, p21 and p53 stainings were inversely related to each other ($P < 0.001$). There was a significant positive association between p21 and AP-2 expression levels ($P = 0.01$). p21 intensity and percentage were higher in Dukes' A and B stages ($P < 0.001$). The cancer-related survival and recurrence-free survival (RFS) rates were significantly lower among patients with a low signal for p21 ($P < 0.001$) and low p21 percentage in tumour epithelium ($P < 0.001$). High p53 staining intensity in tumour epithelium predicted poor survival ($P = 0.01$) and RFS ($P = 0.003$). In the multivariate analysis, p21 percentage distribution independently predicted cancer-related survival in all cases, and p21 expression intensity in T1–4/N0–3/M0 and T1–3/N0/M0 cases. p21 percentage distribution was an independent predictor of RFS in all and T1–3/ N0/M0 cases. AP-2 staining did not reach any prognostic significance. These results suggest that the immunohistochemical detection of cyclin-dependent kinase inhibitor p21 could be used to predict more precisely the outcome of colorectal cancer patients.

**Keywords:** colorectal carcinoma; prognosis; p21; p53; AP-2

p21/WAF1 was first isolated as one of the cyclin-dependent kinase (cdk) interacting proteins induced by wild-type *p53* (Matsushita et al, 1996). The *p53* gene is mutated in a large fraction of cancers, suggesting that the expression of wild-type p53 gene is often rate-limiting for tumour growth (Polyak et al, 1996). Its cell-cycle inhibitory effects are mediated, at least in part, by transcriptional activation of p21. Previous studies (Doglioni et al, 1996; Sasaki et al, 1996) have also revealed differences in p21 and p53 expressions between normal mucosa, adenomas and adenocarcinomas of the large bowel. p21 expression is inversely related to cell proliferation and directly related to terminal differentiation (Doglioni et al, 1996). In addition, high p21 expression has been associated with lower stage (Doglioni et al, 1996; Matsushita et al, 1996; Yasui et al, 1997) and lack of p53 overexpression, with presumed p53 alteration resulting in loss of function (Doglioni et al, 1996).

Activator protein (AP)-2 is a DNA-binding transcription factor that activates p21 expression, providing a link between differentation and negative cell cycle control (Zeng et al, 1997; Gee et al, 1998). A number of genes functioning in ectodermally derived tissues have been reported to be regulated by AP-2. A decrease in AP-2 immunopositivity has been described in cervical intraepithelial neoplasia (CIN) (Hietala et al, 1997). Similarly, in human malignant melanoma, AP-2 expression levels were decreased within tumours compared to strongly immunopositive normal adjacent epidermis (Bar-Eli, 1997). In stage I cutaneous malignant melanoma, reduced AP-2 expression is associated with malignant transformation and tumour progression; furthermore, decreased AP-2 expression is independently associated with elevated risk of subsequent metastatatic behaviour and recurrent disease (Karjalainen et al, 1998).

Prompted by the previously mentioned observations (Bar-Eli, 1997; Hietala et al, 1997; Zeng et al, 1997; Gee et al, 1998; Karjalainen et al, 1998), we hypothesized that AP-2 might up-regulate p21 and function as a tumour suppressor in colorectal carcinoma. Therefore, clinicopathological parameters and prognosis were statistically analysed in relation to p21, p53 and AP-2 protein levels in 162 colorectal carcinoma samples.

### MATERIALS AND METHODS

### Patients

The present study consists of 162 patients diagnosed and treated at Kuopio University Hospital for colorectal adenocarcinoma between 1976 and 1986, and subsequently followed-up for a mean of 14.0 years. These patients were selected from the original cohort of 308 patients from which 146 patients were excluded because adequate histological material for p21, p53 and AP-2 was not available any more. The excluded samples contained either a rather small amount of tumour tissue or there was no tumour left in the section. The clinical staging of all tumours was completed according to UICC (Hermanek and Sobin, 1987) and modified Dukes' classifications (Turnbull et al, 1967). Staging was based on the results of abdominal ultrasonography, bone- and chest

*Received 16 November 1998*
*Revised 9 February 1999*
*Accepted 16 February 1999*

*Correspondence to:* V-M Kosma, Department of Pathology and Forensic Medicine, University of Kuopio, PO Box 1627, FIN-70211 Kuopio, Finland

**Table 1** Clinicopathological data of the patients

| | |
|---|---|
| Number of patients | 162 |
| Mean age of patients (± s.d.) (range) | 65.4 (±11.0) (35.0–88.0) |
| Sex (females/males) | (81/81) |
| Mean (± s.d.) follow-up years | 14.0 (±3.8) |
| Type of primary treatment | |
| Operation | 162 |
| Operation+chemotherapy | 22 |
| Operation+radiotherapy | 17 |
| Surgical treatment | |
| Total colectomy | 6 |
| Hemicolectomy | 40 |
| Anterior resection of rectum | 42 |
| Abdominoperineal resection of rectum | 31 |
| Local excision | 5 |
| Resection of sigmoid | 24 |
| Exploratory laparotomy | 14 |
| T-category: Tis, 1, 2, 3, 4, X | 2, 10, 26, 106, 16, 2 |
| N-category: 0, 1, 2, 3, X | 106, 32, 16, 4, 4 |
| M-category: 0, 1 | 117, 45 |
| Histological grade: 1, 2, 3 | 43, 96, 23 |
| TIL grade: 0, 1, 2, 3 | 10, 100, 44, 8 |
| Dukes': 0, A, B, C, D | 2, 27, 62, 26, 45 |

radiographs, bone scans, computerized tomography, colography, endoscopy (recto-sigmoidoscopy and colonoscopy) and laboratory tests reflecting the possible metastasis. All 162 patients underwent operations and, of these, 22 were additionally treated with chemotherapy and 17 with radiotherapy. The follow-up was done according to the standard practice used in our clinic by the same team of gastroenterologists. The pertinent data of the patients are summarized in Table 1.

### Histology

The tumour samples obtained in the operation were immediately fixed in 10% buffered formalin (pH 7.0), and later embedded in paraffin. Several original sections from each of the primary tumours were re-examined by two observers unaware of the clinical data or the disease outcome, and the most representative tissue block was selected, cut at 5-μm thickness and stained with haematoxylin and eosin (H & E). Tumours were graded as well, moderately, or poorly differentiated (WHO grade). The patients' histopathological data are shown in Table 1.

### Immunohistochemistry and detection of p21, p53 and AP-2

Five-micrometre-thick paraffin sections were rehydrated and washed twice for 5 min with phosphate-buffered saline (PBS). The sections were heated in a microwave oven in 0.05 mol $l^{-1}$ Tris–HCl buffer (pH 9.7) for 2 × 5 min. Endogenous peroxidase activity was blocked with 5% hydrogen peroxide for 5 min, followed with a wash for 2 × 5 min with PBS. The tissue sections were incubated overnight at + 4°C with a p21-specific mouse monoclonal antibody (NCL-WAF-1, Novocastra Laboratories Ltd, UK) at a working dilution of 1:10. Samples were washed twice for 5 min with PBS and incubated for 30 min with biotinylated secondary antibody (Vectastain ABC Elite Kit, Vector Laboratories, Burlingame, CA, USA) in PBS. After two 5-min washings in PBS, the sections were incubated for 40 min in avidin–biotin peroxidase detection solution. Samples were washed 2 × 5 min with PBS, developed with diaminobenzidine tetrahydrochloride (DAB; Sigma, St Louis, MO, USA) for 5 min, slightly counterstained with Mayers's haematoxylin, dehydrated, cleared and mounted with DePex (BDH Supplies, Poole, UK).

The p53 immunoreactivity was demonstrated by means of the same staining protocol. We used a monoclonal DO7 (Dako, Denmark) antibody, known to be specific for both mutant and wild-type forms of p53 protein at a working dilution of 1:1000. For antigen retrieval the samples were boiled in a microwave oven twice for 5 min in citrate buffer (pH 6.0).

The staining protocol for AP-2 has been published previously (Hietala et al, 1997). After eliminating non-specific staining with 1.5% normal goat serum for 30 min, the tissue sections were incubated overnight at +4°C with a primary antibody. We used a rabbit polyclonal AP-2α (C-18) (Santa Cruz Biotechnology, Santa Cruz, CA, USA) antibody, raised against a peptide corresponding to amino acids 420–437 mapping at the carboxy terminus of AP-2 of human origin, at a working dilution of 1:2500. After washings, bound antibody was localized using the same method as described above for p21. In each staining batch a section of human cutaneous malignant melanoma (p21, AP-2) or colorectal carcinoma (p53) with a known positivity for antibodies was used as a positive control for p21, p53 and AP-2. Omission of the antibody served as a negative control.

All slides were evaluated simultaneously with a dual-head microscope (field diameter 490 μm) the observers (KMR and VMK for p21 and AP-2; TP and VMK for p53) being unaware of the clinical data. Disagreement in the assessment of staining was found in fewer than 10% of the slides examined and consensus was reached on further review. The staining intensity of p21, p53 and AP-2 in tumour epithelium and per cent distribution was analysed from the most representative tissue section. p21 and p53 staining intensities in tumour epithelium were scored as follows:

Absent staining was 0
If the staining intensity of tumour cell nuclei was clearly weaker than the positive control, the staining intensity was considered weak (1)
Moderate or strong (2) expression was defined as dark brown staining comparable to the positive control
AP-2 intensity was either negative (0) or weak (1).

For evaluation of AP-2 expression, positive inflammatory cells within the tumour and a section of human cutaneous melanoma were used as positive controls. The fraction of positive tumour cells was primarily analysed in a continuous scale but, for statistical calculations, the percentage distribution of p21 and AP-2 was divided into two groups using median as a cut-off level: ≤ 10%, > 10%, ≤ 30%, > 30% respectively. Finally, the percentage distribution of p53 was scored as previously: ≤ 20%, > 20% (Hirvikoski et al, 1997).

### Analysis of lymphocyte density

The density of tumour-infiltrating lymphocytes (TILs) was graded by two observers as absent, weak, moderate or dense, as described previously (Ropponen et al, 1997). In brief, the TIL level was quantified from ten microscopic fields (×40), and the mean TIL value was calculated. TIL density was classified as absent (grade 0) when < 10 lymphocytes were observed per high-power field (HPF). TIL density was weak (grade 1) when scanty lymphocytes

 © 1999 Cancer Research Campaign

A
B
C

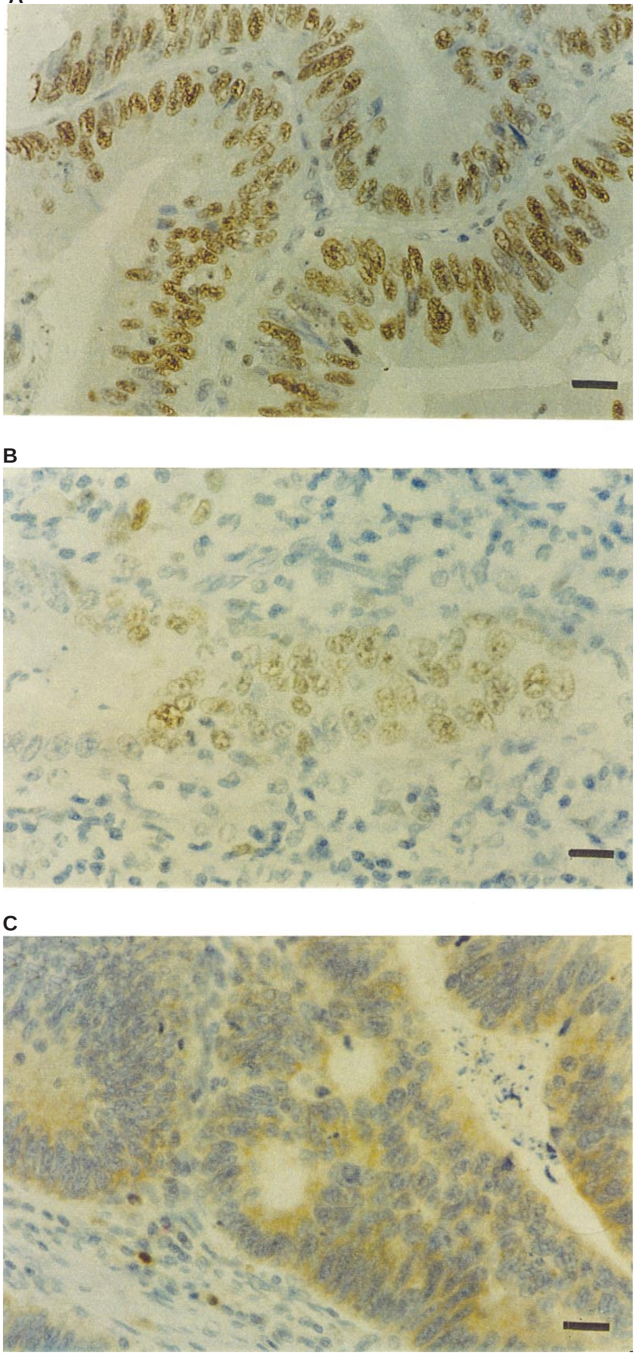

**Figure 1** Immunohistochemical staining for p21 and AP-2 in human colon carcinoma. (**A**) A sample with tumour cells intensely positive for p21. Note the nuclear staining pattern (**B**) A sample with weak nuclear p21 staining intensity in tumour cells. (**C**) A sample with cytoplasmic AP-2 staining in tumour cells. Bar = 60 μm were seen in the stroma and the inflammatory cell reaction around the tumour was mild, i.e. 10–50 lymphocytes per HPF. TIL density was moderate (grade 2) when peri- and/or intra-tumoural lymphocyte infiltrate was intermediate between grades 1 and 3, i.e. lymphocyte level 51–100 per HPF. TIL density was dense (grade 3) when the tumour margins and stroma contained > 100 lymphocytes per HPF.

**Table 2** Expression of p21, p53 and AP-2 in colorectal cancer

| Immunostaining | Intensity (*n*) (%) | Percentage fraction (*n*) (%) |
|---|---|---|
| p21 | 0 (40) (25) | ≤ 10 (81) (50) |
| | 1 (69) (42) | > 10 (81) (50) |
| | 2 (53) (33) | |
| p53 | 0 (8) (5) | ≤ 20 (35) (22) |
| | 1 (67) (42) | > 20 (127) (78) |
| | 2 (87) (53) | |
| AP-2 | 0 (18) (11) | ≤ 30 (74) (46) |
| | 1 (144) (89) | > 30 (88) (54) |

### Mitotic indices

Mitotic figures were counted in the area of the highest mitotic frequency, and ten consecutive high-power microscopic fields (×40) were selected (field diameter 490 μm). The volume-corrected mitotic index (M/V index) method was used in evaluating the mitotic frequency as originally described (Haapasalo et al, 1989). The M/V index expresses the number of mitotic figures per $mm^2$ of neoplastic epithelium in the microscopic image.

### Statistical analysis

In statistical calculations, the SPSS-X programme package was used in an IBM computer. The differences between the means of continuous variables were tested by analysis of variance (ANOVA), and the frequency distributions by the $\chi^2$ test (Mantel and Haenszel, 1992). The univariate survival analysis was based on the life-table method (log-rank analysis) with statistics by Gehan. Cancer-related survival was measured from the date of surgery to the end of follow-up or death. Recurrence-free survival (RFS) was defined as the time elapsed between the primary treatment (date of surgery) and the date of recurrent tumour. Cases with metastases at diagnosis were not included when the RFS was analysed. The causes of death were verified from patient files and death certificates. Multivariate survival analysis (Cox's regression analysis) was performed with the SPSS-X programme package in a step-wise manner (Cox, 1972), and $P \leq 0.05$ and $P \geq 0.01$ were statistically significant for entry and removal limits respectively. Cox's proportional hazard model was used to assess the contribution of the following base-line covariates: age, sex, tumour grade, tumour site (tumours involving rectum $n$ = 78, or not $n$ = 84), Dukes' classification, TNM stage, TILs, p21, p53, AP-2 and M/V index. In the survival analyses, low p53- and p21 intensities (0 and 1) were considered as one group, and moderate and strong intensity (2) was considered as another group.

### RESULTS

### Expression of p21, p53, and AP-2

In normal mucosa adjacent to malignant tissue p21 immunoreactivity was observed mainly in the nuclei of the upper third of the crypts and in the surface epithelium. p53 positivity was not observed in the normal mucosa. AP-2 was expressed weakly in the cytoplasm of normal intestinal epithelium. Twenty-five per cent of the tumours were totally negative for p21, and 33% showed moderate or strong expression intensity. Five per cent of the

*© 1999 Cancer Research Campaign*

**Table 3** Correlation between p21 expression intensity, percentage distribution in tumour epithelium and Dukes' classification, tumour grade and p53

| p21 intensity | 0 *n* (%) | 1 *n* (%) | 2 *n* (%) | p21% (%) (± s.d.) |
|---|---|---|---|---|
| Dukes (*n*) | | | | |
| 0 (2) | 0 (0) | 0 (0) | 2 (4) | 25 (7) |
| A (27) | 1 (3) | 7 (10) | 19 (36) | 30 (22) |
| B (62) | 15 (37) | 21 (30) | 26 (49) | 16 (17) |
| C (26) | 10 (25) | 10 (15) | 6 (11) | 12 (13) |
| D (45) | 14 (35) | 31 (45) | 0 (0) | 8 (10) |
| | $\chi^2 = 50.7$, $P < 0.001$[a] | | | $F = 8.3$, $P < 0.001$[b] |
| Tumour grade (*n*) | | | | |
| 1 (43) | 10 (25) | 14 (20) | 19 (36) | 18 (19) |
| 2 (96) | 24 (60) | 42 (61) | 30 (57) | 14 (18) |
| 3 (23) | 6 (15) | 13 (19) | 4 (7) | 13 (16) |
| | $\chi^2 = 2.42$, $P = 0.11$[a] | | | $F = 0.92$, $P = 0.40$[b] |
| p53 intensity (*n*) | | | | |
| 0 (8) | 5 (13) | 2 (3) | 1 (2) | 4 (7) |
| 1 (67) | 9 (23) | 21 (30) | 37 (70) | 23 (20) |
| 2 (87) | 26 (64) | 46 (67) | 15 (28) | 10 (14) |
| | $\chi^2 = 34.6$, $P < 0.001$[a] | | | $F = 8.3$, $P < 0.001$[b] |

[a]$\chi^2$ test; [b] Analysis of variance.

**Table 4** Correlation between p21, M/V index and AP-2 expression in colorectal carcinoma

| Variables | *n* | Spearman's correlation coefficient | Two-tailed significance |
|---|---|---|---|
| p21 vs. M/V index | 162 | –0.233 | 0.003 |
| p21 vs. AP-2 | 162 | 0.195 | 0.013 |
| p21 vs. p53 | 162 | –0.114 | 0.018 |
| p53 vs. M/V index | 162 | –0.003 | 0.971 |
| p53 vs. AP-2 | 162 | –0.014 | 0.836 |
| AP-2 vs. M/V index | 162 | 0.051 | 0.516 |

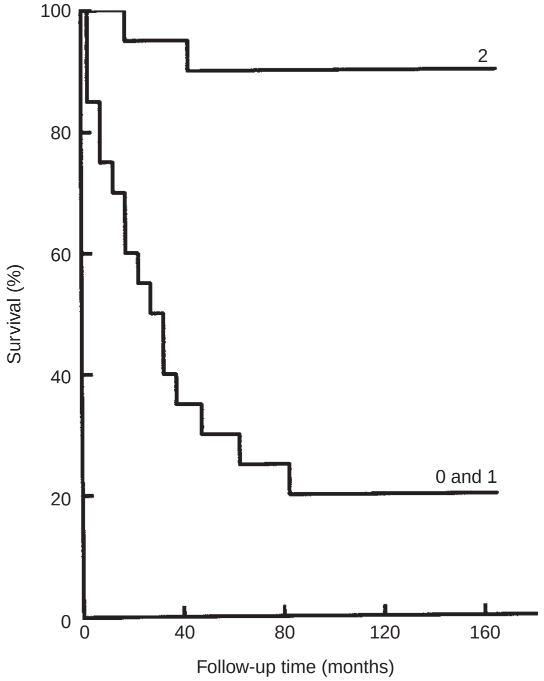

**Figure 2** The survival of 162 patients categorized according to the p21 expression intensity in tumour epithelium. The difference between the curves is significant ($\chi^2 = 48.7$, $P < 0.001$, Curve 0 and 1 = p21 intensity 0 and 1, $n = 109$; curve 2 = p21 intensity 2, $n = 53$)

tumours were totally negative for p53, and 53% showed moderate or strong expression intensity. AP-2 was negative in 11% of the tumours, 89% of the tumours having a weak cytoplasmic AP-2 signal. Seventeen per cent of the tumours had nuclear positivity as well (Figure 1) (Table 2).

### Correlation of p21, p53, and AP-2 to other prognostic factors

The expressions of p53 and p21 correlated inversely. As hypothesized, the AP-2 and p21 expressions were positively associated with each other (Tables 3 and 4). The percentage of p53-reacting cells correlated inversely with p21 intensity (ANOVA, $F = 5.24$, $P = 0.006$). The percentages of p53- and p21-reacting cells were inversely interrelated (ANOVA, $F = 4.23$, $P = 0.04$). There were no significant correlations between tumour grade and p21, p53 or AP-2 expressions. In Dukes' A and B tumours, p21 expression intensity and percentage distribution were higher in tumour epithelium than in Dukes' C and D tumours (Table 3). p53 expression intensity and percentage distribution in tumour epithelium and AP-2 percentage distribution did not correlate with Dukes'. No significant associations were found between p53, AP-2 and TILs or M/V index. p21 expression correlated with M/V index; low p21 signal was associated with high M/V index (Table 4).

### Univariate survival analysis

In univariate survival analysis patients with negative or weak (0 and 1) p21 expression intensity in tumour epithelium had lower survival and RFS rates as compared to patients with moderate or strong p21 expression intensity (2) (Figures 2 and 3). p21 percentage distribution in tumour cells correlated with survival and RFS as well. Patients with over 10% distribution of p21 had higher survival and RFS rate as compared to patients with ≤ 10% distribution of p21 (Figures 4 and 5). Patients with negative or weak (0 and 1) p53 expression intensity in tumour epithelium had both higher survival and RFS rate as compared to patients with moderate or strong p53 expression intensity (2) in their tumour epithelium ($\chi^2 = 5.9$, $P = 0.01$; $\chi^2 = 8.9$, $P = 0.003$ respectively). p53 and AP-2 percentages were not related to cancer-related or RFS (data not shown). In addition, the combination of the results of p21 and p53 expression (p53–/p21–, p53+/p21–, p53–/p21+, p53+/p21+) had no effect on survival or RFS in all patients or in those with Dukes' B stage tumours, probably due to non-comparable group sizes (data not shown).

T1–3/N0/M0 subgroup of patients with moderate or strong p21 expression intensity (2) in tumour epithelium had higher survival rate (90% survived 10 years) as compared to patients with negative or weak p21 expression intensity (0 and 1) in their tumour epithelium (35% survived 10 years) ($\chi^2 = 19.3$, $P < 0.001$). Low p21 expression intensity in tumour epithelium (0 and 1) predicted poorer RFS rate (35% survived recurrence-free 10 years) as compared to patients with moderate or strong p21 expression

© 1999 Cancer Research Campaign

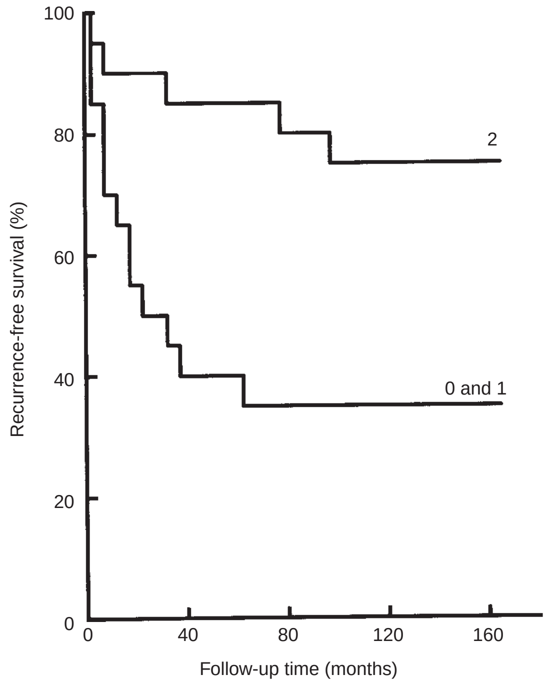

**Figure 3** The recurrence-free survival of 117 patients categorized according to the p21 expression intensity in tumour epithelium. The difference between the curves is significant ($\chi^2$ = 23.9, $P$ < 0.001, Curve 0 and 1 = p21 intensity 0 and 1, $n$ = 64; curve 2 = p21 intensity 2, $n$ = 53)

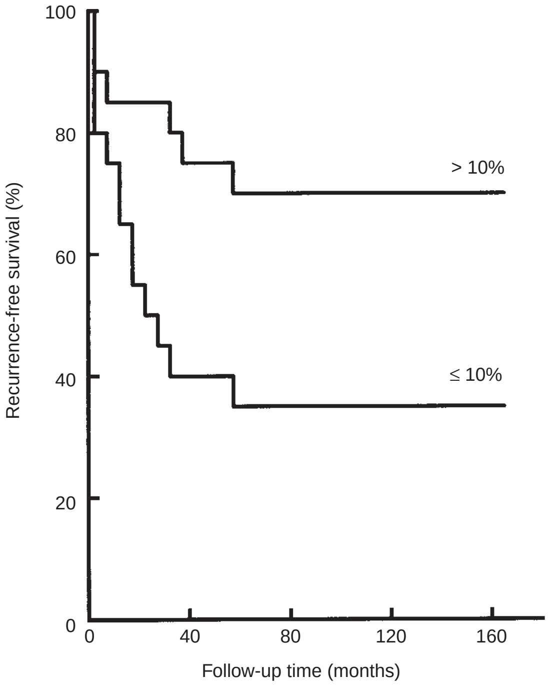

**Figure 5** The recurrence-free survival of 117 patients categorized according to the p21 percentage fraction in tumour epithelium. The difference between the curves is significant ($\chi^2$ = 14.6, $P$ < 0.001, Curve ≤ 10% = p21% ≤ 10, $n$ = 51; curve > 10% = p21% > 10, $n$ = 66)

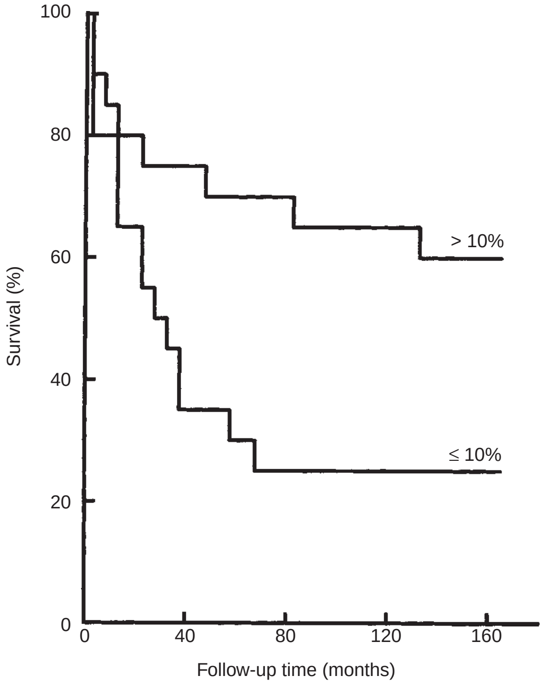

**Figure 4** The survival of 162 patients categorized according to the p21 percentage fraction in tumour epithelium. The difference between the curves is significant ($X^2$ = 12.5, $P$ < 0.001, Curve ≤ 10% = p21% ≤ 10, $n$ = 81; curve > 10% = p21% > 10, $n$ = 81)

intensity (2) in tumour epithelium (80% survived recurrence-free 10 years) ($X^2$ = 14.0, $P$ < 0.001). Similarly patients with low percentage fraction of p21 (≤ 10%) had lower RFS rate (35% survived recurrence-free 10 years) as compared to patients with high percentage fraction of p21 (> 10%) (70% survived recurrence-free 10 years) ($X^2$ = 10.0, $P$ = 0.002). Patients with negative or weak p53 expression intensity in tumour epithelium (0 and 1) survived longer as compared to patients with moderate or strong (2) p53 expression intensity ($X^2$ = 13.5, $P$ = 0.004 and $X^2$ = 4.2, $P$ = 0.04 for RFS).

Chemotherapy or radiation therapy in connection with primary surgical therapy had no effect on survival. Chemotherapy or radiation therapy for treatment of the recurrent disease had no prognostic value since the therapies were given at a very late stage of the disease as a palliative measure. In addition the type of surgery had no effect on survival or RFS.

### Multivariate analysis

The results of multivariate survival analyses are shown in Table 5. In all patients p21 percentage in tumour epithelium predicted survival significantly. In T1–4/N0–3/M0 and T1–3/N0/M0 patients p21 expression intensity and p21 percentage in tumour epithelium significantly predicted survival and RFS respectively. Accordingly, in Dukes' B patients ($n$ = 62) independent prognostic factors of survival were tumour site (patients with their tumours located in colon survived longer) ($P$ = 0.04, relative risk (RR) = 0.36, 95% confidence interval (CI) 0.14–0.95), M/V index ($P$ = 0.009, RR = 1.15, 95% CI 1.04–1.28) and p21 expression intensity in tumour epithelium ($P$ < 0.001, RR = 0.33, 95% CI 0.17–0.63). In addition, significant prognostic factors of RFS in Dukes' B patients ($n$ = 62) were tumour site ($P$ = 0.006, RR = 0.33, 95% CI 0.15–0.73), TILs ($P$ = 0.007, RR = 0.33, 95% CI 0.15–0.73), M/V index ($P$ = 0.03, RR = 1.14, 95% CI 1.01–1.28) and p21 percentage in tumour epithelium ($P$ = 0.01, RR = 0.96, 95% CI 0.92–0.99).

*© 1999 Cancer Research Campaign*

**Table 5** Independent predictors of survival and recurrence-free survival in Cox's analysis. Multivariate analysis included cases with a complete data set available

| Category | β (s.e.) | *P* | Hazard rate (95% CI) |
|---|---|---|---|
| Survival | | | |
| All cases (*n* = 162) | | | |
| Dukes | 0.688 (0.135) | < 0.001 | 1.99 (1.53–2.60) |
| Grade | 0.615 (0.230) | 0.007 | 1.85 (1.18–2.90) |
| p21% | –0.029 (0.011) | 0.01 | 0.97 (0.95–0.99) |
| TILs | –0.643 (0.232) | 0.006 | 0.53 (0.33–0.83) |
| T1–4/N0–3/M0 (*n* = 117) | | | |
| p21 intensity | –0.712 (0.213) | 0.001 | 0.49 (0.32–0.74) |
| M/V index | 0.080 (0.036) | 0.03 | 1.08 (1.01–1.16) |
| N-category | 0.833 (0.256) | 0.001 | 2.30 (1.39–3.80) |
| T1–3/N0/M0 (*n* = 84) | | | |
| Age | 0.075 (0.029) | 0.01 | 1.08 (1.02–1.14) |
| Tumour site | –1.022 (0.474) | 0.03 | 0.36 (0.14–0.91) |
| M/V index | 0.173 (0.055) | 0.002 | 1.19 (1.07–1.32) |
| p21 intensity | –1.105 (0.314) | <0.001 | 0.33 (0.18–0.61) |
| Recurrence-free survival (*n* = 117) | | | |
| T1–4/N0–3/M0 | | | |
| N-category | 0.780 (0.217) | <0.001 | 2.18 (1.43–3.34) |
| p21% | –0.035 (0.011) | 0.001 | 0.97 (0.94–0.99) |
| T1–3/N0/M0 (*n* = 84) | | | |
| p21% | –0.045 (0.014) | 0.001 | 0.96 (0.93–0.98) |

Significance level $P < 0.05$; β = coefficient of the regression model; s.e. = standard error of β; CI = confidence interval

### DISCUSSION

In normal intestinal mucosa, p21 immunoreactivity has been mostly observed in the nuclei of the upper third of the crypts and in the surface epithelium (Doglioni et al, 1996; Sasaki et al, 1996; Yasui et al, 1997), which is in line with our observation. In colorectal and urothelial cancers, p21 expression has been reported to be heterogeneous and decreased compared to normal tissue (Doglioni et al, 1996; Sasaki et al, 1996; Clasen et al, 1998). The majority (75%) of the tumours in our study showed p21 positivity, and 43% expressed p21 moderately or strongly. The overexpression of p21 suppresses tumour growth in several experimental models (Bae et al, 1995; Chen et al, 1995; Li et al, 1995; Tenan et al, 1995). However, p21 overexpression has been associated with higher histological grade in breast cancer and non-small-cell lung cancer (Caffo et al, 1996; Bennett et al, 1998; Rey et al, 1998). In our study, p21 staining did not correlate with tumour grade, which is in accordance with several recent observations in different neoplasms (Doglioni et al, 1996; Slebos et al, 1996; Byrne et al, 1997). Overexpression of p21 has also been observed in low-grade (I–II) urothelial tumours (Clasen et al, 1998). In addition, Doglioni et al (1996), Wang et al (1997) and Yasui et al (1997) described higher p21 expression in stages 0–2 colon carcinomas than in stages 3 and 4 carcinomas. There is also evidence supporting the hypothesis that p21 may exert its tumour suppressive effects in the early stages of tumour development (Ogawa et al, 1997; Clasen et al, 1998) to which the present study gives further strengthening. However, some cancer studies have not revealed any association between tumour stage and p21 expression (Slebos et al, 1996; Byrne et al, 1997; Bennet et al, 1998). Taking together the available information it is obvious that additional data are needed to explain p21's multiplex functions more precisely during cancer progression.

The relative p21 mRNA levels have been suppressed in tumours with p53 mutations, indicating the necessicity of wild-type p53 for p21 activation (Matsushita et al, 1996). In the present study, p21 overexpression correlated with low p53 level (by DO7 antibody), which is in line with other recent papers (Doglioni et al, 1996; Sasaki et al, 1996). Doglioni et al (1996) have suggested that high p21 expression could be related to normal or increased p53 function, and low p21 expression could be related to inactivation of p53 function. This, in other words, represents p53-dependent p21 induction pathway. Similar results were found in breast cancer by Ellis et al (1997) and Bukholm et al (1997). Interestingly, DNA-based mutational analysis did not reveal mutations to be responsible for the changed regulatory effects of p53 in their study (Ellis et al, 1997). It is important to remember that p53 overexpression by immunohistochemistry may not necessarily represent mutated p53, and furthermore, mutated p53 may still retain some trancriptional activity (Doglioni et al, 1996).

Elsewhere p21 induction has been observed to be p53-independent in breast cancer (Rey et al, 1998), ovarian cancer (Elbendary et al, 1996; Anttila et al, 1999) and in human leukaemia (Zhang et al, 1995). Our results suggested a p53-independent mechanism of p21 activation only in a subgroup of patients (Table 3). This kind of phenomenon (independent and dependent mechanisms) has also been observed by Yasui et al (1997) in their series of colorectal tumours. In addition, previous studies on endometrial (Backe et al, 1997; Ito et al, 1997), ovarian (Werness et al, 1997) and urothelial carcinoma (Clasen et al, 1998) have failed to show a correlation between p21 expression and p53. The significance of intratumoural p21 and p53 staining heterogeneity can be hypothesized as follows: a population of cancer cells with one functional copy of p53 might still be able to activate p21 within a tumour in which the other cells have already lost both functional copies. Accordingly, p21 is weakly expressed in cells with one functional allele of p53, and it is absent in cells with a mutation or loss of both alleles (Sasaki et al, 1996). From previous studies it is known that the immunoreactivity of p53 parallels the degree of mutation, and weak staining of p53 indicates the presence of both wild-type and mutant p53 proteins (Ohue et al, 1994). Based on the above cited reports it is reasonable to suggest that p21 activation by p53 is complex, tissue-specific and may vary in different phases of cell differentiation. Macleod et al (1995) showed that many adult tissues expressed high levels of p21, including thymus, brain and intestine as compared to liver, muscle and kidney. It is possible that other cyclin–cdk inhibitors such as p27 are functioning in these tissues to induce growth arrest prior to differentiation. Alternatively these tissues may have reached a stable maintenance of growth arrest (Macleod et al, 1995). Our results showed no p53 protein expression in epithelial cells of normal colorectal mucosa, suggesting that p21 gene expression may occur by p53-independent pathways in differentiated cells. This finding supports the observation by Parker et al (1995) that increased levels of p21 may also be linked with terminal differentiation of specialized cells.

Induction of p21 is thought to result in growth inhibition of human cancer cells (Zeng et al, 1997). Different isoforms of transcription factor AP-2 have been shown to activate the expression of both positive and negative growth regulators, including c-erbB-2 and p21$^{WAF1/CIP1}$. In addition, in vitro and in vivo studies have suggested that AP-2 may have the ability to restrict tumour growth (Bar-Eli, 1997; Karjalainen et al, 1998). The strongest clinical evidence for AP-2's tumour suppressive properties comes from the study by Karjalainen et al (1998). While studying malignant

© 1999 Cancer Research Campaign melanomas they were able to show that AP-2 expression progressively decreased as the tumour thickness increased (Karjalainen et al, 1998). Decreasing AP-2 expression was also a strong predictor for poor clinical outcome (Karjalainen et al, 1998). Further evidence for AP-2's importance in cancer cell regulation is derived from breast cancer studies where c-erbB-2 has been shown to be regulated by AP-2 (Bosher et al, 1995). In this case overexpression of c-erbB-2 is widely thought to contribute to poor clinical outcome (Bosher et al, 1995). However, in the present series of colorectal carcinoma, AP-2 staining did not reach prognostic significance. Taken together, the data available so far suggest that the effects of AP-2 are diverse and that different isoforms of AP-2 have different tissue specificity.

In the current study, p21 proved to be an antiproliferative factor; it inversely correlated with mitotic index. Similarly, an inverse correlation between p21 and Ki-67 proliferation antigen has been documented recently in colorectal and endometrial cancer (Doglioni et al, 1996; Palazzo et al, 1997). On the other hand, several studies have not been able to demonstrate p21's antiproliferative action (Backe et al, 1997; Diab et al, 1997; Werness et al, 1997). Accordingly, in breast cancer and malignant melanoma increased cell proliferation has been positively related to p21 expression (Rey et al, 1998; Karjalainen et al, 1999). Possible explanations in these cases include transient p21 expression before entry into S phase, abnormal function of p21, or that tumour cells have become refractory to inhibitory p21 signals.

Newly recognized cell cycle regulators were tested for their relevance in predicting patients' clinical outcome in the present study. Strong intensity and high percentage distribution of p21 in tumour epithelium predicted longer survival and RFS time. In the multivariate analysis, p21 expression intensity and percentage distribution were independent prognostic factors for overall survival and RFS. M/V index also proved to be a significant indicator of survival in T1–4/N0–3/M0 and T1–3/N0/M0 cases. Our results give further support for the previously documented role of p21 as a negative growth regulator in colorectal cancer (Doglioni et al, 1996; Sasaki et al, 1996; Yasui et al, 1997). p21 may inhibit cancer spreading by suppressing abnormally accelerated cell cycle, and may therefore be a good candidate for a new therapeutic approach to control tumour cell growth. However, before being ready for clinical testing, further studies explaining p21's possible cell type specificity are needed.

### ACKNOWLEDGEMENTS

We thank Mrs Aija Ruotsalainen and Aija Parkkinen for technical assistance and Ms Eeva Oittinen for secretarial assistance with the manuscript. This study has been supported by grants from the Cancer Fund of North Savo (Savon Syöpärahasto), and by EVO-funding of Kuopio University Hospital.

### REFERENCES

Anttila MA, Kosma V-M, Hongxiu J, Puolakka J, Juhola M, Saarikoski S and Syrjänen K (1999) p21/WAF1 expression as related to p53, cell proliferation and prognosis in epithelial ovarian cancer. *Br J Cancer* **79**: 1870–1878

Backe J, Gassel AM, Hauber K, Krebs S, Bartek J, Caffier H, Kreipe HH, Muller-Hermelink HK and Dietl J (1997) p53 protein in endometrial cancer is related to proliferative activity and prognosis but not to expression of p21 protein. *Int J Gynecol Pathol* **16**: 361–368

Bae I, Fan S, Bhatia K, Kohn KW, Fornace AJ Jr and O'Connor PM (1995) Relationships between G1 arrest and stability of the p53 and p21cip 1/waf1 proteins following gamma-irradiation of human lymphoma cells. *Cancer Res* **55**: 2387–2393

Bar-Eli M (1997) Molecular mechanisms of melanoma metastasis. *J Cell Physiol* **173**: 275–278

Bennett WP, El-Deiry WS, Rush WL, Guinee DG, Freedman AN, Caporaso NE, Welsh JA, Jones RT, Borkowski A, Travis WD, Fleming MV, Trastek V, Pairolero PC, Tazelaar HD, Midthun D, Jett JR, Liotta LA and Harris CC (1998) p21 WAF/CIP1 and transforming growth factor β1 protein expression correlate with survival in non-small cell lung cancer. *Clin Cancer Res* **4**: 1499–1506

Bosher JM, Williams T and Hurst H (1995) The developmentally regulated transcription factor AP-2 is involved in c-erbB-2 overexpression in human mammary carcinoma. *Proc Natl Acad Sci USA* **92**: 744–747

Bukholm IK, Nesland JM, Kåresen R, Jacobsen U and Borresen AL (1997) Relationship between abnormal p53 protein and failure to express p21 protein in human breast carcinomas. *J Pathol* **181**: 140–145

Byrne RL, Horne CHW, Robinson MC, Autzen P, Apakama I, Bishop RI, Neal DE and Hamdy FC (1997) The expression of waf-1, p53 and bcl-2 in prostatic adenocarcinoma. *Br J Urol* **79**: 190–195

Caffo O, Doglioni C, Veronese S, Bonzanini M, Marchetti A, Buttita F, Fina P, Leek R, Morelli L, Palma PD, Harris AL and Barbareschi M (1996) Prognostic value of p21/WAF1 and p53 expression in breast carcinoma: an immunohistochemical study in 261 patients with long-term follow-up. *Clin Cancer Res* **2**: 1591–1599

Chen YQ, Cipriano SC, Arenkiel JM and Miller FR (1995) Tumor suppression by p21/WAF1. *Cancer Res* **55**: 4536–4539

Clasen S, Schulz WA, Gerharz C-D, Grimm M-O, Cristoph F and Schmitz-Dräger BJ (1998) Frequent and heterogenous expression of cyclin-dependent kinase inhibitor WAF1/p21 protein and mRNA in urothelial carcinoma. *Br J Cancer* **77**: 515–521

Cox DR (1972) Regression models and life tables with discussion. *J Roy Stat Soc B* **34**: 187–192

Diab SG, Yu YY, Hilsenbeck SG, Allred DC and Elledge RM (1997) WAF1/CIP1 protein expression in human breast tumours. *Breast Cancer Res Treat* **43**: 99–103

Doglioni C, Pelosio P, Laurino L, Macri E, Meggiolaro E, Favretti F and Barbareschi M (1996) p21/WAF1/CIP1 expression in normal mucosa and in adenomas and adenocarcinomas of the colon: its relationship with differentiation. *J Pathol* **179**: 248–253

Elbendary AA, Cirisano FD, Evans AC Jr, Davis PL, Iglehart JD, Marks JR and Berchuck A (1996) Relationship between p21 expression and mutation of the p53 tumor suppressor gene in normal and malignant ovarian epithelial cells. *Clin Cancer Res* **2**: 1571–1575

Ellis PA, Lonning PE, Borresen-Dale A, Aas T, Geisler S, Akslen LA, Salter I, Smith IE and Dowsett M (1997) Absence of p21 expression is associated with abnormal p53 in human breast carcinomas. *Br J Cancer* **76**: 480–485

Gee MS, Sarkisian CJ and El-Deiry WS (1998) Identification of a novel AP-2 consensus DNA binding site. *Biochem Biophys Res Commun* **243**: 307–316

Haapasalo H, Collan Y, Atkin NB, Pesonen E and Seppä A (1989) Prognosis of ovarian carcinomas: prediction by histoquantitative methods. *Histopathology* **15**: 167–178

Hermanek P and Sobin LH (eds) (1987) *TNM Classification of Malignant Tumours*, 4th edn. Springer: Berlin

Hietala KA, Kosma V-M, Syrjänen KJ, Syrjänen SM and Kellokoski JK (1997) Correlation of MIB-1 antigen expression with transcription factors Skn-1, Oct-1, AP-2, and HPV type in cervical intraepithelial neoplasia. *J Pathol* **183**: 305–310

Hirvikoski P, Kumpulainen E, Virtaniemi J, Johansson R, Haapasalo H, Marin S, Halonen P, Helin H, Raitiola H, Pukander J, Kellokumpu-Lehtinen P and Kosma VM (1997) p53 expression and cell proliferation as prognostic factors in laryngeal squamous cell carcinoma. *J Clin Oncol* **15**: 311–3120

Ito K, Sasano H, Matsunaga G, Sato S, Yajima A, Nasim S and Garret C (1997) Correlations between p21 expression and protein alterations, and survival in patients with endometrial carcinoma. *J Pathol* **183**: 318–324

Karjalainen J, Kellokoski J, Eskelinen M, Alhava E and Kosma V-M (1998) Downregulation of transcription factor AP-2 predicts poor survival in stage I cutaneous malignant melanoma. *J Clin Oncol* **16**: 3584–3591

Karjalainen JM, Eskelinen MJ, Kellokoski JK, Reinikainen M, Alhava EM and Kosma V-M (1999) p21WAF1/CIP1 expression in stage I cutaneous malignant melanoma its relationship with p53, cell proliferation and survival. *Br J Cancer* **79**: 895–902

Li YJ, Laurent-Puig P, Salmon RJ, Thomas G and Hamelin R (1995) Polymorphisms and probable lack of mutation in the waf-cip 1 gene in colorectal cancer. *Oncogene* **10**: 599–601

© 1999 Cancer Research Campaign 

Macleod KF, Sherry N, Hannon G, Beach D, Tokino T, Kinzler K, Vogelstein B and Jacks T (1995) p53-dependent and independent expression of p21 during cell growth, differentiation, and DNA damage. *Genes Dev* **9**: 935–944

Mantel N and Haenszel W (1992) Statistical aspects of the analysis of data from retrospective studies of disease. *J Natl Cancer Inst* **51**: 556–561

Matsushita K, Kobayashi S, Kato M, Itoh Y, Okuyama K, Sakiyama S and Isono K (1996) Reduced messenger RNA expression level of $p21^{CIP1}$ in human colorectal carcinoma tissues and its association with p53 gene mutation. *Int J Cancer (Pred Oncol)* **69**: 259–264

Ogawa M, Maeda K, Onoda N, Chung Y-S and Sowa M (1997) Loss of $p21^{WAF1/CIP1}$ expression correlates with disease progression in gastric carcinoma. *Br J Cancer* **75**: 1617–1620

Ohue M, Tomita N, Monden T, Fujita M, Fukunaga M, Takami K, Yana I, Ohnishi T, Enomoto T, Inoue M, Shimano T and Mori T (1994) A frequent alteration of p53 gene in carcinoma in adenoma of colon. *Cancer Res* **54**: 4798–4804

Palazzo JP, Mercer WE, Kovatich AJ and McHugh M (1997) Immunohistochemical localization of p21 (WAF/CIP1) in normal, hyperplastic, and neoplastic uterine tissues. *Hum Pathol* **28**: 60–66

Parker SB, Eichele G, Zhang P, Rawls A, Sands AT, Bradley A, Olson EN, Harper JW and Elledge SJ (1995) p53-independent expression of $p21^{Cip1}$ in muscle and other terminally differentiating cells. *Science* **267**: 1024–1027

Polyak K, Waldman T, He TC, Kinzler KW and Vogelstein B (1996) Genetic determinants of p53-induced apoptosis and growth arrest. *Genes Dev* **10**: 1945–1952

Rey MJ, Fernandez PL, Jares P, Munoz M, Nadal A, Peiro N, Nayach I, Mallofre C, Muntane J, Campo E, Estape J and Cardesa A (1998) p21 WAF/Cip 1 is associated with cyclin $D1^{CCND1}$ expression and tubular differentiation but is independent of p53 overexpression in human breast carcinoma. *J Pathol* **184**: 265–271

Ropponen KM, Eskelinen MJ, Lipponen PK, Alhava E and Kosma V-M (1997) Prognostic value of tumour infiltrating lymphocytes (TILs) in colorectal cancer. *J Pathol* **182**: 318–324

Sasaki K, Sato K, Kurose A and Ikeda E (1996) Immunohistochemical detection of $p21^{WAF1/CIP1/SDI1}$ and p53 proteins in formalin-fixed, paraffin-embedded tissue sections of colorectal carcinoma. *Hum Pathol* **27**: 912–916

Slebos RJC, Baas IO, Clement M, Polak M, Mulder J-W, van den Berg FM, Hamilton SR and Offerhaus GJA (1996) Clinical and pathological associations with p53 tumour-suppressor gene mutations and expression of $p21^{WAF1/Cip1}$ in colorectal carcinoma. *Br J Cancer* **74**: 165–171

Tenan M, Carrara F, Di Donato S and Finocchiaro G (1995) Absence of mutations and identification of two polymorphism in the SSCP and sequence analysis of p21CKI gene in malignant gliomas. *Int J Cancer* **62**: 115–117

Turnbull RB, Kyle K, Watson FB and Spratt J (1967) Cancer of the colon: the influence of the no-touch isolation technique on survival rates. *Ann Surg* **166**: 400–427

Wang A, Yoshimi N, Ino N, Tanaka T and Mori H (1997) WAF1 expression and p53 mutations in human colorectal cancers. *J Cancer Res Clin Oncol* **123**: 118–123

Werness BA, Jobe JS, DiCioccio A and Piver MS (1997) Expression of the p53 induced tumor suppressor $p21^{waf1/cip1}$ in ovarian carcinomas: correlation with p53 and Ki-67 immunohistochemistry. *Int J Gynecol Pathol* **16**: 149–155

Yasui W, Akama Y, Yokozaki H, Semba S, Kudo Y, Shimamoto F and Tahara E (1997) Expression of $p21^{WAF1/CIP1}$ in colorectal adenomas and adenocarcinomas and its correlation with p53 protein expression. *Pathol Int* **47**: 470–477

Zhang W, Grasso L, McClain CD, Gambel AM, Cha Y, Salvatore T, Deisseroth AB and Mercer WE (1995) p53-independent induction of WAF1/CIP1 in human leukemia cells is correlated with growth arrest accompanying monocyte/macrophage differentiation. *Cancer Res* **55**: 668–674

Zeng Y-X, Somasundaram K and El-Deiry WS (1997) AP2 inhibits cancer cells growth and activates $p21^{WAF1/CIP1}$ expression. *Nat Genet* **15**: 78–82

 © 1999 Cancer Research Campaign
